# 

**Published:** 1893-11

**Authors:** 


### Society Meetings.

THE New York State Association of Railway Surgeons will hold its third annual meeting, under the presidency of Dr. George Chaffee, at the New York Academy of Medicine, on Wednesday, November 15, 1893. A cordial invitation is extended to the profession.

---

THE Southern Surgical and Gynecological Association will hold its sixth annual meeting at New Orleans, La., on Tuesday, Wednesday, and Thursday, November 14, 15, and 16, 1893, under the presidency of Dr. Bedford Brown, of Alexandria, Va.

The good quality of work which this Association does, warrants the expectation that the attendance will, as usual, be very large this year.

### BOOKS RECEIVED.

Index-Catalogue of the Library of the Surgeon-General's Office, United States Army. Authors and Subjects, Volume XIV. Sutures-Universal. Quarto, pp. 14—1016. Washington: Government Printing Office. 1893.

Sander's Question Compends, No. 12. Essentials of Minor Surgery, Bandaging, and Venereal Diseases, arranged in the form of questions and answers, prepared especially for students of medicine. By Edward Martin, A. M., M. D., Clinical Professor of Genito-Urinary Diseases; Instructor in Operative Surgery and Lecturer on Minor Surgery, University of Pennsylvania; Surgeon to the Howard Hospital; Assistant Surgeon to the University Hospital, etc., etc. Second edition, revised and enlarged, seventy-eight illustrations. Philadelphia: W. B. Sanders, 1893.

Transactions of the Medical Association of Georgia: Forty-fourth Annual Association, 1893. Octavo, pp. 426. Atlanta, Ga.: Published by the Association. 1893.

Hernia: Its Palliative and Radical Treatment in Adults, Children, and Infants. By Thomas H. Manley, A. M., M. D., Visiting Surgeon to Harlem Hospital; Consulting Surgeon to Fordham Hospital; Member of the New York Academy of Medicine, American Medical Association, New York State and County Medical Associations, International Medical Congress, Pathological Society, National Association of Railway Surgeons, etc., etc. Octavo, pp. 231. Philadelphia: The Medical Press Company, Limited. 1893.

A Treatise on the Science and Practice of Midwifery. By W. S. Playfair, M. D., F. R. C. P., Professor of Obstetric Medicine in King's College, London; Examiner in Midwifery to the Universities of Cambridge and London, and to the Royal College of Physicians. Sixth American from the eighth English edition. Edited, with additions, by Robert P. Harris, M. D. In one octavo volume of 697 pages, with 217 engravings and five plates. Cloth, $4.00; leather, $5.00. Philadelphia: Lea Brothers & Co. 1893.

A Handbook of Ophthalmic Science and Practice. By Henry E. Juler, F. R. C. S., Ophthalmic Surgeon to St. Mary's Hospital; Surgeon to the Royal Westminster Ophthalmic Hospital, London. New (second) edition, revised and enlarged. In one handsome octavo volume, of 562 pages, with 201 engravings, seventeen colored plates, test-types, and color blindness test. Cloth, $5.50; leather, $6.50.

Supplement to the Reference Handbook of the Medical Sciences. By Various Writers. Illustrated by chromo-lithographs and fine wood-engravings. Edited by Albert H. Buck, M. D., New York City. Volume IX. Imperial octavo, 1084 pages. Cloth, price, $6.00; sheep, price, $7.00; half morocco, price, $8.00. New York: William Wood & Company.

Minor Surgery and Bandaging. By Henry R. Wharton, M. D., Demonstrator of Surgery in the University of Pennsylvania. In one 12mo volume of 529 pages, with 416 engravings, many being photographic. Cloth, $3.00. Philadelphia: Lea Brothers & Co. 1893.

A Text-Book of Ophthalmology. By William F. Norris, M. D., Professor of Ophthalmology in the University of Pennsylvania, and Charles A. Oliver, M. D., Surgeon to Wills Eye Hospital, Philadelphia. In one very handsome octavo volume of 641 pages, with 357 engravings and five colored plates. Cloth, $5.00; leather, $6.00. Philadelphia: Lea Brothers & Co. 1893.

Anatomy, Descriptive and Surgical. By Henry Gray, F. R. S., Lecturer on Anatomy at St. George's Hospital, London. New American from the thirteenth enlarged and improved English edition. Edited by T. Pickering Pick, F. R. C. S., Examiner in Anatomy, Royal College of Surgeons of England. In one imperial octavo volume of 1100 pages, with 635 large engravings. Price, with illustrations in colors, cloth, $7.00; leather, $8.00. Price, with illustrations in black, cloth, $6.00; leather, $7.00. Philadelphia: Lea Brothers & Co. 1893.

